# The Expansion of Genetic Testing in Cardiovascular Medicine: Preparing the Cardiology Community for the Changing Landscape

**DOI:** 10.1007/s11886-023-02003-4

**Published:** 2024-01-26

**Authors:** Nosheen Reza, Raye L. Alford, John W. Belmont, Nicholas Marston

**Affiliations:** 1 Division of Cardiovascular Medicine, Department of Medicine, Perelman School of Medicine at the University of Pennsylvania, Philadelphia, PA, USA; 2 Houston, TX, USA; 3 Department of Medicine, Brigham and Women’s Hospital, Harvard Medical School, Boston, MA, USA

**Keywords:** Genetics, Genetic testing, Cardiovascular genetics, Inherited heart disease

## Abstract

**Purpose of Review:**

Pathogenic DNA variants underlie many cardiovascular disease phenotypes. The most well-recognized of these include familial dyslipidemias, cardiomyopathies, arrhythmias, and aortopathies. The clinical presentations of monogenic forms of cardiovascular disease are often indistinguishable from those with complex genetic and non-genetic etiologies, making genetic testing an essential aid to precision diagnosis.

**Recent Findings:**

Precision diagnosis enables efficient management, appropriate use of emerging targeted therapies, and follow-up of at-risk family members. Genetic testing for these conditions is widely available but under-utilized.

**Summary:**

In this review, we summarize the potential benefits of genetic testing, highlighting the specific cardiovascular disease phenotypes in which genetic testing should be considered, and how clinicians can integrate guideline-directed genetic testing into their practice.

## Introduction

Our understanding of cardiovascular genetics has grown dramatically in recent decades, and with significantly reduced cost and increased availability of clinical genetic testing has come the reality of incorporating genetics into routine practice. However, the uptake of diagnostic genetic testing in the cardiology community has been slow and has primarily been limited to specialized genetics centers at leading academic institutions. In combination with low literacy in cardiovascular genetics among cardiologists [1], this has created an environment in which single-gene cardiovascular diseases frequently go undiagnosed. As Dr. Eugene Braunwald stated in a lecture titled *Directions of Cardiology in the Next Decade: 2022–2032*, “the principal role of the cardiologist in 2022 is to recognize and manage established disease, but by 2032 that role will require the interpretation and application of genetic information for the prevention, diagnosis, and treatment of cardiovascular disease” [2]. As the field of cardiovascular genetics and its impact on patient care expands, so too must the genetic knowledge and infrastructure within the cardiology community.

In this review, we aim to provide a summary of clinical cardiovascular genetics for the practicing cardiologist, including the clinical advantages of genetic testing, clinical scenarios for which it should be considered, and the available options to perform testing when clinically indicated.

## Advantages of Genetic Testing

Cardiovascular genetics is a rapidly evolving specialty of cardiovascular medicine that has not historically been addressed in traditional cardiology fellowship training [3]. As a result, many cardiologists may still view genetics as primarily a research endeavor or academic exercise that will not impact practice. However, it is estimated that 6–10% of the population is or will be affected by a genetic disorder in their lifetimes [4]. While some of these conditions first present in childhood, patients with genetic cardiac disease frequently experience their first symptoms in adulthood [5, 6]. Much recent research has demonstrated that there are important genetic components in common diseases but for most patients the genetic contributions are polygenic — the accumulation of hundreds, thousands, or millions of small effects. There is progress in identifying individuals who have a polygenic risk that is comparable to that from strongly causative rare genetic variants. The latter are called Mendelian or monogenic disorders because they follow clearer rules of inheritance in families. This review focuses on monogenic disorders because testing for them is clinically available, although we believe that diagnostic testing for polygenic causes will be available in the future. Monogenic disorders occur in every cardiovascular disease subspecialty, and so it is expected that all cardiology clinicians will encounter genetically mediated disease in their practices.

**Fig. 1:**
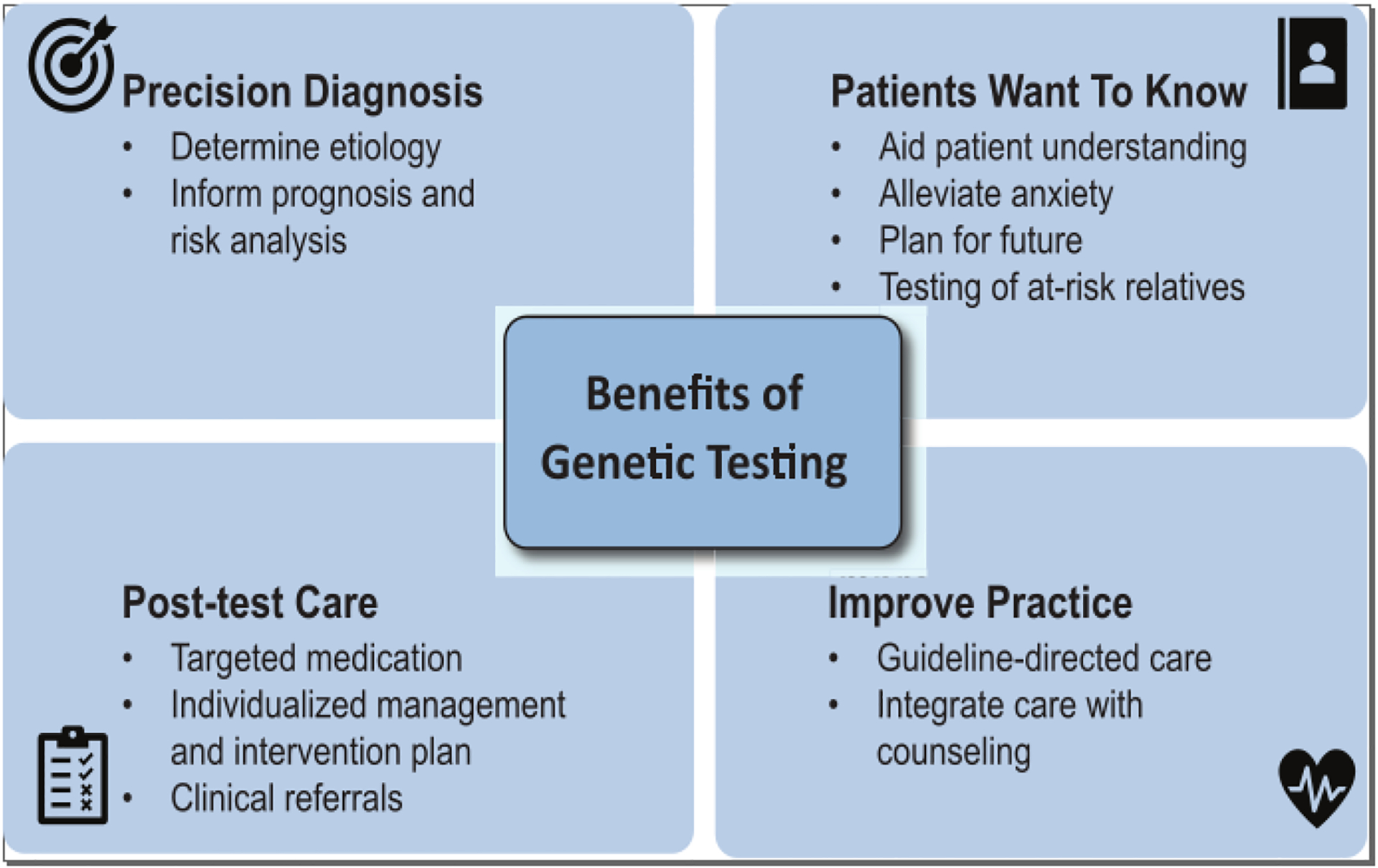
Clinical utilities of cardiovascular genetic testing. Genetic testing supports physician diagnostic and therapeutic decision making and adds important new information of use to patients and their families

There are several fundamental advantages to establishing a molecular diagnosis through genetic testing (Fig. 1):

1. *Patients with a cardiac phenotype want to know the cause of disease*: In adults, risk factors for cardiovascular disease, such as smoking and physical inactivity, are typically thought to be acquired. A confirmed genetic diagnosis can often relieve anxiety or guilt [7].
2. *Genetic testing can provide a diagnosis*: Many abnormal cardiovascular phenotypes have acquired and inherited causes, and genetic testing can provide etiology of disease. In a single-center observational study of 2472 adult patients who underwent septal myectomy for severe symptomatic obstructive hypertrophic cardiomyopathy (HCM), almost 1 in 5 patients had an alternate histopathologic diagnosis that could have been diagnosed by genetic testing, like amyloidosis and Fabry disease [8].
3. *Knowing a patient’s genotype can provide prognostic information*: For example, patients with confirmed familial hypercholesterolemia (FH) are threefold more likely to have cardiovascular events than their non-FH counterparts with the same LDL-C level [9]. In patients with HCM, those with disease-causing variants in sarcomere genes had twofold greater risk for adverse outcomes such as arrhythmias and heart failure compared to those without these genetic variants [5]. This knowledge can inform shared decision-making between patients and clinicians.
4. *A molecular diagnosis can change management*: For many clinicians, this is the strongest argument for pursuing genetic testing, as it directly impacts patient care. Examples of this are highlighted in Table 1. In the case of patients with FH, the primary prevention guidelines recommend lower LDL-C targets, which may mean initiating more aggressive lipid-lowering therapies that would not have otherwise been considered [10]. Insurance coverage of these therapies may even be more easily achieved with genotype-confirmed FH. Moreover, genetic panel testing can also detect rare genetic variants that if present can significantly alter management. For example, an expanded dyslipidemia genetic testing panel sequences genes that are causal for FH pheno-copy conditions, such as sitosterolemia, which is caused by an overabsorption of dietary plant sterols. Making this diagnosis greatly changes the approaches to treatment, as the first-line lipid-lowering therapy is ezetimibe rather than statins, and patients are advised to limit many healthy foods such as nuts, avocados, and olive oil.
5. *Identification of a disease-causing genetic variant triggers cascade testing and can facilitate reproductive planning*: Beyond the diagnostic, prognostic, and management benefits of genetic testing for the individual patient, testing results can have a ripple effect throughout families, potentially identifying previously unrecognized disease in additional individuals. This multiplier effect significantly increases the yield of genetic testing, as each test can have downstream benefits for multiple individuals within a family. For those family members carrying the variant of interest, the relevant clinical workup is performed to assess for the presence of preclinical disease. This early identification of genetic risk and subclinical manifestation of disease can have significant implications, such as exercise restriction and ICD implantation in the case of some inherited cardiomyopathies and arrhythmias [11]. However, there is potential disutility or even harm associated with familial cascade testing. Individuals should be consented with acknowledgement of the right not to know about the presence of a genetic disorder. Prognosis for either the occurrence of cardiomyopathy or significant complications is uncertain when testing is carried out in unaffected relatives. Some individuals may feel increased anxiety as a consequence of the testing or they may be concerned about insurance discrimination. Testing children should be undertaken cautiously with the concern for direct benefit during childhood and, when appropriate, leaving open the option for choosing testing when they become adults.

## Who to Consider for Genetic Testing?

**Fig. 2:**
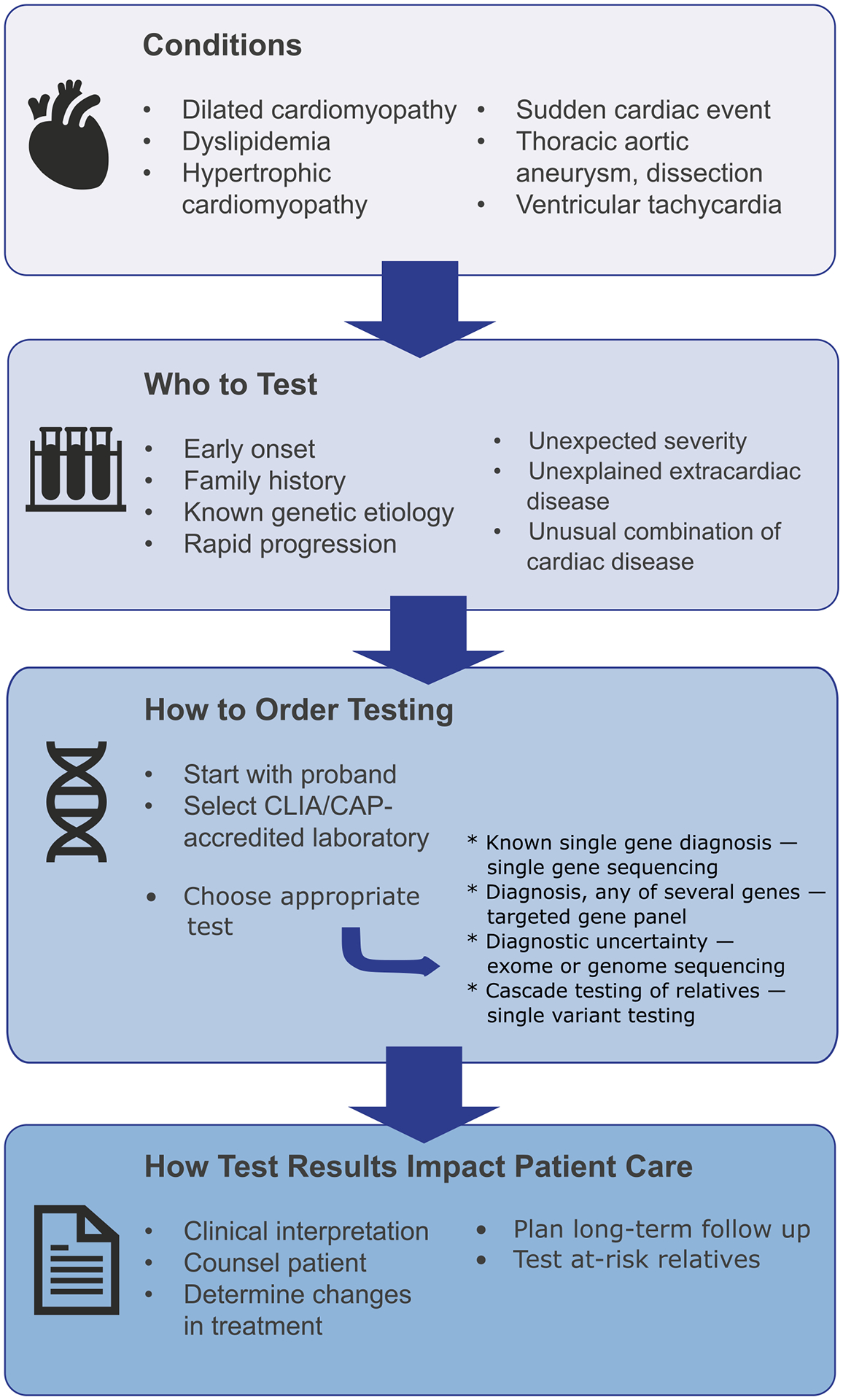
The genetic testing process. Selection of patients for testing is the most impactful step in the diagnostic process. Resources are available to help with test selection and the clinical interpretation of the test results

Once the advantages of genetic testing are established, the next step is to identify who should undergo testing. In general, patients with disease of early onset and unexpected severity should raise suspicion of a genetic condition, especially in the presence of a family history of similar disorders (Fig. 2). Additionally, unusual combinations of symptoms or unexplained extracardiac disease can suggest the presence of a syndrome or systemic genetic disease. While many cardiovascular conditions may have a genetic component, there are several that should raise clinical suspicion for a primary genetic etiology and prompt consideration for the evaluation of monogenic disease. We have outlined six of the most common phenotypes below, each of which are included in US and European guideline statements addressing recommendations for genetic testing in cardiovascular disease [12, 13••, 14]. Table 1 summarizes these cardiovascular phenotypes, their genetic differential diagnosis, the relevant genes for each condition, and how genetic testing for each phenotype can impact patient management.

### 1. Hypertrophic cardiomyopathy:

Hypertrophic cardiomyopathy (HCM) is a common cardiovascular condition, with a prevalence of 1 in 200 to 500 individuals [15]. It is characterized by asymmetric myocardial hypertrophy, most commonly in the septum but other variants exist. Variation in eight sarcomeric genes accounts for the majority of HCM. Disease-causing variants in the genes *MYH7* and *MYBPC3* are responsible for approximately 80% of familial HCM, and variants in other genes such as *TNNI3, TNNT2, TPM1, MYL2, MYL3*, and *ACTC1* each account for 1–5% of patients [15]. Among patients with the HCM phenotype, gene panel testing identifies a pathogenic or likely pathogenic mutation in approximately 30% of sporadic and 50–60% of familial cases [16]. Other genetic causes of the left ventricular hypertrophy phenotype are also included on the typical HCM gene testing panel and include transthyretin amyloidosis, glycogen storage diseases, and Anderson-Fabry disease. Non-genetic causes of left ventricular hypertrophy include hypertensive heart disease, aortic stenosis, and athlete’s heart, and there can be phenotypic overlap with these conditions and sarcomeric HCM. In the 2020 American Heart Association/American College of Cardiology Guideline for the Diagnosis and Treatment of Patients with Hypertrophic Cardiomyopathy, genetic testing has a Class 1 recommendation to confirm the genetic bases of disease and to identify family members at risk for developing HCM [15].

### 2. Dilated cardiomyopathy:

Dilated cardiomyopathy (DCM) affects up to 1 in 250 individuals in the general population, and patients with nonischemic dilated cardiomyopathy comprise 40–50% of participants in heart failure clinical trials [6]. Among those cases classified as “idiopathic,” familial DCM, occurring when two closely related family members meet diagnostic criteria for idiopathic DCM, accounts for 20% [16]. The genetic etiologies of familial DCM are quite heterogeneous, with more than 30 genes implicated in the pathogenesis [17]. Gene curation studies have demonstrated that 19 genes have strong or moderate evidence to support their causal relationship with DCM [18]. Titin (*TTN*) is the most common gene implicated in DCM, representing 15–20% of DCM cases, followed by Lamin A/C (*LMNA*) cardiomyopathy at 6% [16]. *MYH6* (4%), *MYH7* (4%), and *TNNT2* (3%) are sarcomeric genes that are associated with both DCM and HCM [17]. DCM can also be seen in several muscular dystrophy syndromes, often in combination with skeletal muscle involvement [19–21]. Identifying a monogenic cause of familial DCM can impact evaluation, risk stratification, prognosis, and treatment [22–25].

### 3. Ventricular arrhythmias:

Ventricular arrhythmias in the absence of established coronary artery disease, active myocarditis, or severe left ventricular dysfunction of known etiology should prompt consideration of a broad differential. The many potential genetic etiologies can be subdivided into variants affecting the myocardium and electrical conduction. Arrhythmias arise in primary myocardial diseases, including HCM and DCM as discussed above, as well as arrhythmogenic and non-compaction cardiomyopathies. Inherited arrhythmia syndromes affecting the conduction system include inherited long-QT syndrome, Brugada syndrome, and catecholaminergic polymorphic ventricular tachycardia. Identifying the causative gene may affect the treatment recommendation. As an example, it is thought that beta-blockers are less effective in long-QT syndrome 3, which is caused by gain-of-function pathogenic variants in *SCN5A* (sodium channel protein type 5 subunit alpha, NaV1.5), compared to other forms of long-QT syndrome, and mexiletine may be added in such cases [13••, 26]. Mexilitine is an inhibitor of the sodium channel and has been shown to shorten QTc, reduce the duration of ventricular repolarization, and reduce life-threatening arrhythmias in patients with LQT3 [27].

### 4. Severe dyslipidemias/premature atherosclerosis:

Coronary artery disease is common in older age, but premature myocardial infarction (MI) should prompt consideration of genetically mediated atherosclerosis if no other clear triggers are present. This is most often related to familial dyslipidemias such as familial hypercholesterolemia [28, 29, 30•], another very common cardiovascular genetic condition occurring at similar rates as familial HCM. The likelihood of FH increases significantly with earlier ages of MI and is present in approximately 1 in 5 patients with MI under the age of 45 (according to FH Foundation) [31]. The standard FH panel includes four genes, with *LDLR* (low-density lipoprotein receptor) mutations representing approximately 85% of cases. Expanded dyslipidemia panels are also available and include genes responsible for FH phenocopies such as sitosterolemia and lysosomal acid lipase deficiencies. In the absence of premature coronary disease, significantly elevated lipid levels should also raise suspicion for familial dyslipidemias. LDL-C levels > 190 mg/dl are often used as a threshold for possible heterozygous FH; however, the higher the levels the more likely an FH variant will be identified, and very high levels (often > 500 mg/dl) suggest homozygous FH [32]. Prior to FH testing, it can be helpful to use either the Dutch or Simon Broome diagnostic criteria [28, 29, 30•] which incorporates personal and family history along with LDL-C level and exam findings to provide the likelihood of FH. Genetic testing is often required to make a definitive diagnosis of FH, which carries lower LDL-C targets, supports earlier and more aggressive lipid lowering, and often improves medication compliance [13••]. Beyond LDL-C, genetics can also cause elevation in other lipid markers that carry important clinical consequences. Severe hypertriglyceridemia, especially levels > 880 mg/dl (10 mmol/L) should prompt consideration of genetic causes. Familial hypertriglyceridemia, familial combined hyperlipidemia, and familial chylomicronemia syndrome (FCS) are also tested on expanded dyslipidemia panels, the latter of which has approved therapies available in Europe and novel agents under investigation in phase 2/3 clinical trials. Lipoprotein(a) is a genetically mediated LDL-like particle that carries cardiovascular risk but is not lowered with statins. Checking serum levels of Lp(a) is now recommended once during adulthood by many national societies, and genetic testing does not currently play a role [33–35].

### 5. Aortopathies:

Heritable forms of thoracic aortic aneurysm and dissection represent another major category of cardiogenetic disorders. There are 19 genes implicated in familial aortopathy, 11 of which are well established as having high penetrance [36]. New gene associations with aortopathy continue to be identified and as evidence accumulates many more monogenic forms may ultimately be reportable. Thoracic aortic aneurysms are often related to connective tissue diseases, such as Marfan syndrome, Ehlers-Danlos, Loeys-Dietz, and mixed connective tissue disorders. Therefore, a thorough physical exam is critical when examining patients with aortic aneurysm or prior dissection. Those who do have non-cardiac findings or syndromic features will often have an identifiable pathogenic variant [37]. But it is important to note that many patients with genetically determined aortopathy do not have syndromic features and genetic testing should be considered in all patients with thoracic aortic aneurysm especially if there is a family history, presentation at a young age, rapid progression, dissection at a diameter less than 5 cm, or co-occurrence of coarctation or atrial septal defect [38–44]. Identifying a familial cause of thoracic aneurysm has important clinical implications as some conditions are associated with greater risk of rupture and warrant earlier surgical intervention [13••].

### 6. Sudden cardiac arrest and death:

The differential diagnosis for sudden cardiac arrest is broad, and genetic etiologies should always be considered. Each of the five phenotypes listed above can cause sudden cardiac arrest, and thus, comprehensive phenotyping with electrocardiogram, cardiovascular imaging, and coronary evaluation are critical to identify the potential etiology. Depending on this evaluation, genetic testing may be indicated. Postmortem examination may be negative or ambiguous. Since life-threatening arrhythmias can occur in both genetic cardiomyopathies and inherited arrhythmias, combination genetic panels can be ordered for cases of sudden cardiac death without a clear etiology.

In the future, many cardiovascular diseases may become treatable with gene or even variant-specific therapies designed to replace or modulate impaired physiological or cellular functions. Monoclonal antibodies [45], allele-specific oligonucleotides [46], and gene therapy [47–49] are among the targeted methods that may find a place in the treatment of genetically determined cardiovascular diseases.

## How to Order Genetic Testing

If a cardiovascular genetic disorder is suspected, genetic testing should be initiated with the family proband, ideally the person in the family who is the most severely affected or has the earliest age of onset (Fig. 2). Once an appropriate proband is identified and agreeable to genetic testing, the next step is the testing process. This includes selecting the type of test (i.e., gene panel testing, single-gene or single-variant testing, whole-exome or -genome sequencing), the accredited laboratory that will perform and report the test, and the medium that will be submitted for testing (i.e., blood versus saliva). Because of the striking differences in technical platforms and their performance, genetic test selection can seem difficult. Next-generation sequencing (NGS) gene panels are currently the most commonly used method because of their relatively low cost and good clinical sensitivity. We have included a comparison table (Supplementary Table 1) for readers who would like more detail. Involvement of a genetic counselor to facilitate pre- and post-test genetic counseling is crucial to facilitate patients’ and families’ understanding of complex genetic and medical information.

Genetic testing for diagnosis of patients with suspected inherited cardiovascular diseases should always be carried out in an accredited clinical laboratory. In the USA, all labs providing genetic test results should be accredited by the College of American Pathologists and the test certified by the Clinical Laboratory Improvement Amendments (CLIA) program. In the EU, labs and tests should be accredited in their jurisdiction. Almost all clinical tests for cardiovascular disease have been developed within the lab offering them (called laboratory-developed tests or LDTs). LDTs must be validated, and documentation must be available for the certifying organizations. Typically, genetic tests that are sold directly to consumers are not subject to these regulations and should not be used for diagnosis or screening.

When a clinician has high suspicion for a disease caused by only one gene, then a single-gene test can be used. However, there is often diagnostic uncertainty, and genetic testing is not used exclusively for confirmation but rather to cover a broader differential diagnosis—gene panels are effective for this. Some gene panels may only sequence a few genes, e.g., for familial hypercholesterolemia, but many currently available gene panels are much broader with tens or hundreds of genes included. In the situation of a clear clinical phenotype, e.g., severe hypercholesterolemia, clinicians should select the testing strategy that most narrowly captures the genes likely to be involved. However, when there is phenotypic uncertainty or overlap, e.g., cardiomyopathy combined with arrhythmia, a broader testing strategy with a larger gene panel may be of higher utility. Larger panels do not uniformly increase the rate of confirmed diagnoses, and sequencing a larger number of genes increases the likelihood that a variant of unknown/uncertain significance, which may not be the cause of disease, will be found [50]. Another consideration is the type of genetic variant that is suspected to be causal in a particular patient. Rare single nucleotide variants, which are detected by most tests, are the most common cause of monogenic disease, but it is also clear that some patients have difficult-to-detect copy number and structural variants. Array-based tests, for example, efficiently identify copy number variations but are limited in their ability to detect the broadest range of small sequence variants. Currently, exome and genome sequencing are not yet routine in adult cardiovascular clinical care and are more often used in pediatric and syndromic conditions. Because genome sequencing can detect a wider array of types of genetic abnormalities like small copy number variants and repeat expansions, genetic testing laboratories may use this as a platform technology in the future as costs shift from sequencing to interpretation.

### Genetic Test Availability

Navigating the various genetic testing offerings can be confusing for clinicians. The Genetic Testing Registry, supported by the National Library of Medicine, provides a central clearing-house for lab and test information. The most important considerations in selecting a testing laboratory are whether the genetic test being offered includes the genes most relevant for the phenotype being evaluated, whether there are restrictions or preferences for the lab given the patient’s insurance coverage, and evidence that the laboratory is using the best standards for interpretation and reporting.

In recent years, the ClinGen consortium has established a pragmatic framework for ranking the evidence for gene-disease relationships [36, 51, 52•, 53]. Lab tests should at least cover genes with definitive, strong, and moderate support for disease association. Lab tests may include other genes, but these should be interpreted cautiously and reports that contain variants of unknown significance should ideally lead to a referral to a specialist with deep expertise on the particular gene and disease. Specialized cardiogenetic clinics are being established in many tertiary care centers and offer expertise in pre- and post-test genetic counseling and test interpretation. Pre-test genetic counseling importantly incorporates information on the effects of a genetic diagnosis on health insurance coverage and the psychosocial aspects of genetic disease. Patients and clinicians alike should recognize that a negative genetic test result does not eliminate the possibility of a genetic condition.

An excellent and near comprehensive list of genetic testing laboratories can be found at the NIH Genetic Testing Registry https://www.ncbi.nlm.nih.gov/gtr/.

## Challenges of Genetic Testing

Genetic testing presents several broad challenges that are not unlike other complex medical tests. The first challenge is to relate the patient’s clinical presentation to the decision about which test to select. Patients with similar clinical presentations can have distinct genetic causes, making it difficult for the cardiologist to predict which gene is involved. Phenotype overlap is closely related to the problem of locus heterogeneity in which the same genetic disorder can be caused by pathogenic DNA variants in several different genes. Gene panels are constructed so that the physician is not forced to guess which gene is causative in a particular patient.

The second challenge is to interpret test results with pathogenic variants given uncertainties about potential future clinical impact. Variable expressivity describes the range of clinical features and severity associated with disease-causing genes. For example, finding a molecular diagnosis of Emery-Dreifuss muscular dystrophy may increase the probability of complications, like an arrhythmia, but not be determinative. Incomplete penetrance (not all individuals with a pathogenic DNA variant may be clinically affected) is another consideration when testing relatives of a patient with a known molecular diagnosis. Like non-genetic risk factors where duration of exposure affects the final risk, a major factor affecting penetrance is age.

Another challenge is that DNA variants not previously observed in patients can be quite challenging to interpret. The lab must try to group the novel variant with other variants where the interpretation is more secure. The American College of Medical Genetics and Genomics has established a scoring system for the interpretation of variant pathogenicity and sets out recommendations for reporting. The scoring system has been adapted by cardiovascular genetics specialists to tailor the weighting of functional and predictive evidence for known cardiovascular disease genes. Nevertheless, variants of uncertain significance (VUS) are often reported because the clinical presentation is consistent with genes in the test panel. We caution physicians that all test reports require clinical judgement and evidence integration. Variant classifications reflect such judgements in the lab as well as individual lab reporting policies. Most specialized genetic cardiovascular centers address these complex issues with all specialists (cardiologists, medical geneticists, molecular biologists, bioinformatician, etc.) within multidisciplinary cardiovascular genome boards. Thus, the need for a close multidisciplinary collaboration with genetic centers is for the time being essential to ensure adequate patient care.

Finally, negative test results do not rule out genetic disorders. It is important to continue regular cardiac evaluation in first-degree family members when proband genetic testing does not reveal a pathogenic variant. Essentially the negative predictive value of genetic tests is still unknown and perhaps unmeasurable because of the different possible underlying genetic mechanisms. Clinical follow-up is still required for probands and relatives who test negative. If the proband has a positive genetic test (where there is a pathogenic or likely pathogenic variant that can be related to their clinical disease), then a negative result in a close relative does substantially reduce the probability that they would be affected by the suspected genetic disorder. Negative genetic tests in probands do need regular reevaluation as genetic testing options may change over time.

## Potential Barriers to Genetic Testing

The primary patient concerns about genetic testing center around discrimination, privacy, and cost. In the USA, the Genetic Information Nondiscrimination Act (GINA) bars health insurance companies from using genetic results to deny health insurance. Although the protections conferred by GINA do not extend to life, disability, or long-term care insurance, there is state-by-state and employer-based variability in protections conferred through other legal mechanisms. The National Human Genome Research Institute maintains freely available resources regarding genetic discrimination (https://www.genome.gov/about-genomics/policy-issues/Genetic-Discrimination). The primary EU law that prohibits genetic discrimination is the General Data Protection Regulation (GDPR) with particular implications for research [54]. The GDPR prohibits the use of genetic data without explicit consent. The EU has also adopted the Charter of Fundamental Rights, which prohibits discrimination based on genetic features [55]. Some EU member states have enacted their own laws specifically aimed at preventing genetic discrimination [56]. For example, The Dutch Medical Examination Act (MEA) restricts insurers and employers in using genetic test results for decisions on employment or insurance [57].

Another common concern regarding genetic testing is cost, although this has become less of a barrier in recent years. Probands typically incur the highest costs for testing, which can range from less than $100 to a few thousand dollars depending on the genetic test order, testing laboratory, and insurance coverage plan. Most health insurance plans cover genetic testing for specific conditions; preauthorization may be required. Genetic testing laboratories have various policies regarding cascade testing that can alleviate testing costs for family members. Consultation with a medical geneticist and/or genetic counselor may be helpful in obtaining insurance coverage in some circumstances.

Beyond testing itself, access to trained professionals with knowledge in cardiovascular genetics, e.g., genetic counselors, is another barrier to streamlined comprehensive genetic care. The National Society of Genetic Counselors has created a searchable directory that offers access to over 3300 genetic counselors in the USA, including those who meet with patients via phone, video conferencing, and other virtual methods [58]. In practices that do not have dedicated genetic counseling services, this may be an option that can be employed by practices. As recognition of inherited cardiovascular diseases expands, infrastructure to support genetic counseling and testing will increasingly need to be a consideration for clinicians and health systems.

## Future Directions in CV Genetics

### Broader Education in the CV Community

Genetic testing is an underutilized aid to the diagnosis of patients with cardiovascular disorders. Increasing patient awareness of the contribution of genetics to cardiovascular disease may lead them to ask their physicians about the utility of genetic testing in their care. Ongoing education of the cardiovascular community is the key to increase clinician awareness of the benefits of genetic testing in clinical care [59]. This will need to include increased emphasis in cardiovascular scholarship and media, dedicated local and national continuing medical education initiatives, and integration of more robust cardiovascular genetics exposure in our cardiovascular medicine training programs.

### Beyond Monogenic Inheritance

The future may also bring new approaches to genetic testing, such as the incorporation of polygenic risk scores into clinical practice. In contrast to monogenic disorders where a single variant or mutation can be disease causing, polygenic variation represents hundreds, thousands, or even millions of variants with small effect sizes on a given chronic cardiovascular condition. When each of these small effect variants is added together, the result is called a polygenic risk score, which can be used to assign patients a percentile of the genetic risk that they carry for a given condition such as coronary artery disease, atrial fibrillation, or stroke. For those at the very highest spectrum of polygenic risk, their probability of developing disease is similar to that of monogenic conditions [60]. Polygenic risk score testing is available for direct-to-consumer use but has not yet made its way into clinical practice guidelines. However, a recent statement from the AHA on polygenic risk score testing highlights the progress that has been made and how their role in clinical care will likely evolve over time [60].

## Conclusion

Expanded implementation of genetic testing in cardiovascular care provides an opportunity to make precision diagnoses that have implications on prognosis, management, and health of family members. Currently, genetic testing is viewed as a highly specialized process reserved for cardiovascular genetic specialists. However, given the population prevalence and breadth of cardiovascular phenotypes for which genetic testing is warranted, clinical cardiologists should be able to recognize the role of genetics in clinical care, identify the indications for genetic testing, and be aware of the resources to order testing. This will take time to implement throughout the healthcare system, but it begins with educating cardiologists and will ultimately end with better care for our patients.

## Supplementary Material

Supplementary Material

## Figures and Tables

**Table 1:** Examples of gene-directed management and therapeutic decision making*

Phenotype	Genetic diagnoses	Genes of interest	Potential clinical implications of genetic testing in each phenotype
Hypertrophic cardiomyopathy	Familial HCM	*MYBPC3*, *MYH7*, *TNNI2*, *TNNI3*, *TPM1*, *ACTC1, MYL2*, *MYL3*	Myosin modulators; Mavacamten [61, 62] Tafamidis [63, 64], inotersen [65] and patisiran [66] Enzyme replacement with recombinant alpha-glucosidase [67] Enzyme replacement with agalsidase beta [68] Management guidelines [69, 70] Clinical trials [70] Cascade screening
	TTR amyloid	*TTR*	
	Glycogen storage disease	GSD II and III	
	Fabry disease	*GLA*	
	Noonan syndrome/RASopathies	*PTPN11, SOS1, RAF1, KRAS, NRAS, SHOC2, CBL, PTPN11, RAF1, RASA1, HRAS, BRAF, MAP2K1, MAP2K2, KRAS*	
Dilated cardiomyopathy	Titin cardiomyopathy	*TTN*	Arrhythmia monitoring [23, 71] Increased risk of atrial and ventricular arrhythmias Corticosteroids Cascade screening
	Lamin cardiomyopathy	*LMNA*	
	Other familial DCM	*MYH7, TNNT2, BAG3, RBM20, TNNC1, TNNI3, TPM1, SCN5A, PLN*	
	Duchenne/Becker muscular dystrophy	*DMD*	
	Emery-Dreifuss muscular dystrophy	*EMD*, *LMNA*, *FHL1* (X-linked)	
	Myotonic dystrophy	*DMPK*, *CNBP*	
	Limb-girdle muscular dystrophy	*CAPN3*, *DYSF*	
	Friedreich ataxia	*FXN*	
Ventricular arrhythmia and sudden cardiac death	ARVC	*PKP2, DSP, DSC2, TMEM43, DSG2*	Beta-blockers Exercise restrictions Implantable defibrillator Ablation Cascade screening
	Brugada syndrome	*SCN5A*	
	Long-QT syndrome	*KCNQ1, KCNH2, SCN5A*	
	Short-QT syndrome	*KCNQ1, KCNH2, KCNJ2, SLC4A3*	
	CPVT	*RYR2*	
	WPW	*PRKAG2*	
	LV non-compaction	*TAZ*	
	DCM	*LMNA, RBM20, PLN, FLNC, EMD*	
	HCM	*MYH7, MYBPC3*	
Severe dyslipidemias/premature atherosclerosis	Familial hypercholesterolemia	*LDLR, APOB, PCSK9, LDLRAP1*	High dose statins; PCSK9 inhibition [9, 10] Ezetimibe; cholestyramine Varying dietary recommendations [72] Volanesorsen [73] Clinical trials [74–77] Cascade screening
	Sitosterolemia	*ABCG5, ABCG8*	
	FCS/familial hypertriglyceridemia	*LPL, APOA5, APOC2, GPD1, GPIHPB1, LMF1*	
Aortopathies	Marfan syndrome	*FBN1*	Beta-blockers and ARBs [78] Irbesartan Differing thresholds for valve intervention Cascade screening
	Ehlers-Danlos syndrome (EDS)	*COL5A1, COL5A2, COL1A1, COL3A1, TNXB, PLOD1, COL1A2, FKB14, ADAMSTS2*	
	Loeys-Dietz syndrome (LDS)	*TGFβR1, TGFβR2, SMAD3, TGFβ1, TGFβ2*	
	Arterial tortuosity syndrome	*SLC2A10*	
	Congenital Arachnodactly (Beale’s Syndrome)	*FBN2*	
	Familial thoracic aortic aneurysms and aortic dissections (TAAD)	*ACTA2, FLNA, MYH11, MYLK, NOTCH1, PRKG1, BGN* (and genes listed above)	

* Gene lists and implications are intended to be conceptually illustrative; this is not intended to represent a comprehensive list of gene-disease associations or therapeutic strategies
