# Supplementary material for: The Expansion of Genetic Testing in Cardiovascular Medicine: Preparing the Cardiology Community for the Changing Landscape
Source: Curr Cardiol Rep. 2024 Jan 26;26(3):135–46. doi: 10.1007/s11886-023-02003-4 (PMC10990779; doi:10.1007/s11886-023-02003-4)
Supplement: Supplementary file 1 — Supplementary file1 (DOCX 19.8 KB) [file 11886_2023_2003_MOESM1_ESM.docx]

Supplementary Table 1. A comparison of different clinical genetic testing platforms

| **Platform** | **Technology** | **SNV, Indel^1^** | **CNV^2^** | **Mito^3^** | **Repeat Expansion^4^** | **Non-coding and Structural** | **Primary Use Cases** | **Limitations** |
| --- | --- | --- | --- | --- | --- | --- | --- | --- |
| SNP or CGH array | Hybridization^5^ | ✓ | ✓ |  |  |  | Detection of CNV; common variant polygenic scores | Limited clinical sensitivity; low utility of current PRS |
| PCR | Amplification |  |  |  | ✓ |  | Friedreich’s Ataxia; Myotonic Dystrophy | Clinical diagnosis |
| Single Gene | Sanger | ✓ |  |  |  |  | Known familial mutation; specific clinical suspicion e.g. Fabry’s, TTR amyloidosis | Genetic heterogeneity |
| Gene Panel | NGS-panel^6^ | ✓ |  |  |  |  | Cardiomyopathies; inherited arrhythmias; aortopathies; dyslipidemias | Limited or no support for CNV and other variant types; VUS |
| Exome | NGS-exome^7^ | ✓ | ✓ | ✓ |  |  | Non-specific and syndromic presentations | Cost, VUS |
| Genome | NGS-genome^8^ | ✓ | ✓ | ✓ | ✓ | ✓ | Comprehensive testing of most clinically important variant types | Cost and clinical test availability |

^1^ SNV – single-nucleotide variant; Indel – small insertion, deletion, or combination insertion/deletion

^2^ CNV – copy-number variant

^3^ Mito – mitochondrial variants, genes, or genome

^4^ Repeat expansion – diseases caused by unstable small DNA repeats; usually neurological but in the case of Friedreich ataxia and myotonic dystrophy cardiac involvement can be clinically significant

^5^ Hybridization to oligonucleotides on solid phase or bead arrays followed by fluorescent detection by various chemistries

^6^ NGS-panel – next generation sequencing applied to gene panels; most commonly sequencing-by-synthesis but several variations are applied to clinical testing; usually gene exons are targeted by either capture or amplification

^7^ NGS-exome – protein-coding DNA captured by liquid phase hybridization before sequencing

^8^ NGS-genome – unbiased DNA preparation allowing for coverage of gene and non-genic elements; facilitates analysis of almost all variant types. In research, novel chemistries allow very long segments of DNA to be analyzed.
